# Frailty transitions predict healthcare use and Medicare payments in older Mexican Americans: a longitudinal cohort study

**DOI:** 10.1186/s12877-020-01583-y

**Published:** 2020-06-01

**Authors:** Chih-Ying Li, Soham Al Snih, Lin-Na Chou, Amol Karmarkar, Yong-Fang Kuo, Kyriakos S. Markides, Kenneth J. Ottenbacher

**Affiliations:** 1 Department of Occupational Therapy, University of Texas Medical Branch, 301 University Blvd., Galveston, TX 77555-1142 USA; 2 Division of Rehabilitation Sciences, University of Texas Medical Branch, 301 University Blvd., Galveston, TX 77555-1137 USA; 3 Sealy Center on Aging, University of Texas Medical Branch, 301 University Blvd., Galveston, TX 77555-0137 USA; 4 Department of Preventive Medicine & Community Health, University of Texas Medical Branch, 301 University Blvd., Galveston, TX 77555-0137 USA

**Keywords:** Frailty, Elderly, Healthcare cost, Minority health, Diversity, Mexican Americans

## Abstract

**Background:**

Little is known regarding the impact of transitions in frailty on healthcare use and payment in older Mexican Americans. We address this gap in knowledge by investigating the effect of early transitions in physical frailty on the use of healthcare services and Medicare payments involving older Mexican Americans.

**Methods:**

Longitudinal analyses were conducted using the Hispanic Established Populations for the Epidemiological Study of the Elderly (Hispanic-EPESE) survey data from five Southwest states linked to the Medicare claims files from the Centers for Medicare and Medicaid Services. Seven hundred and eighty-eight community-dwelling Mexican Americans 72 years and older in 2000/01 were studied. We used a modified Frailty Phenotype (unintentional weight loss, weakness, self-reported exhaustion and slow walking speed) to classify frailty status (non-frail, pre-frail or frail). Each participant was placed into one of 5 frailty transition groups: 1) remain non-frail, 2) remain pre-frail, 3) remain frail, 4) improve (pre-frail to non-frail, frail to non-frail, frail to pre-frail) and 5) worse (non-frail to pre-frail, non-frail to frail, pre-frail to frail). The outcomes for the one-year follow-up period (2000–2001) were: (a) healthcare use (hospitalization, emergency room [ER] admission and physician visit); and (b) Medicare payments (total payment and outpatient payment).

**Results:**

Mean age was 78.8 (SD = 5.1) years and 60.3% were female in 1998/99. Males who remained pre-frail (Odds Ratio [OR] = 3.49, 1.13–10.8, remained frail OR = 6.92, 1.61–29.7) and transitioned to worse frail status (OR = 4.49, 1.74–11.6) had significantly higher hospitalization risk compared to individuals who remained non-frail. Males in the ‘worsened’ groups, and females in the ‘improved’ groups, had significantly higher Medicare payments than individuals who remained non-frail (Cost Ratio [CR] = 2.00, 1.30–3.09; CR = 1.53, 1.12–2.09, respectively].

**Conclusions:**

Healthcare use and Medicare payments differed by frailty transition status. The differences varied by sex. Research is necessary to elucidate the relationship between frailty transitions and outcomes, sex difference and Medicare payment for older Mexican Americans living in the community.

## Background

Frailty is a common geriatric condition resulting from age-related physical and mental decline often associated with adverse health complications such as falls, disability, hospitalization and mortality [1–6]. The prevalence of frailty is high in community-dwelling older adults, with 10–25% for those ≥65 years, 46% for those ≥80 years and 65% of those ≥90 years [2, 7]. The impact and prevalence of frailty varies across racial and ethnic groups and is influenced by many factors, including social determinants, access to care and the presence of comorbid conditions (e.g., diabetes) [5, 7–9]. The focus of our study is physical frailty in older Mexican Americans. Mexican Americans and other Latinos are the fastest growing older population in the U.S. and are expected to double by 2050 compared to Non-Hispanic Whites and African Americans [10].

Frailty transition is characterized by moving in and out of the stages of frailty [11–14]. Previous studies [15–20] found that baseline frailty status, age, sex and length of follow-up are associated with frailty transition patterns. Overall, investigators have reported that up to 43% of community-dwelling non-disabled older adults transitioned to a worse state of frailty, compared to 20–23% improving, during a 12–54 month follow-up period [15, 19–21]. Lee et al. [19] found that more females than men had frailty improvement (26.6% versus 23.4%) after 48 month follow-up, but this advantage in females is not consistent in the literature. Previous studies have also found that persons > 70 years of age reporting poor quality of life with slow gait speed, diabetes and low education transitioned more rapidly to poor frailty status [14, 22].

The impact of frailty on healthcare use and expenditures (including payment and cost) has received increasing attention [23–34]. Being frail is associated with greater healthcare use, including inpatient, post-acute, outpatient, skilled nursing and long-term or curative care [23–29]. Being frail is also associated with increased healthcare expenditures, such as ambulatory health-care costs [30], outpatient care cost [26], overall hospital care cost [31, 32] and surgical care cost [33, 34]. However, the associations between the change of frailty status and healthcare use or expenditures remains largely unknown.

A better understanding of the dynamic nature of the biological, physical, social and environmental factors associated with frailty transitions in older Mexican Americans will help us develop treatment programs and preventive strategies for frailty. The purpose of our study was to advance knowledge regarding the transition process of physical frailty from non-frail to pre-frail and frail; and to determine the level of healthcare resources and payments associated with these transitions in older Mexican Americans living in the community. We hypothesized based on previous research [23–34] and our clinical experience that transitions to a worse frailty status will be associated with more healthcare use and increased Medicare payments compared to other frailty transitions.

## Methods

### Data source

We linked longitudinal survey data from the Hispanic Established Populations for the Epidemiological Study of the Elderly (Hispanic EPESE) to the Medicare claims files from the Centers for Medicare and Medicaid Services (CMS). The Hispanic EPESE is an ongoing longitudinal study that has tracked the health and activity of community-dwelling older Mexican Americans since 1993. Nine waves of data from 1993 to 2017 have been collected. Participants were randomly selected using geographic probability sampling procedures from five states: Texas, Colorado, New Mexico, Arizona and California. Detailed descriptions of the Hispanic EPESE can be found elsewhere [9, 35]. Data from the Hispanic EPESE is archived and available from the National Archive of Computerized Data on Aging [36].

Medicare files were obtained from the CMS from January 1, 1999 to December 31, 2012. These files were linked to participants enrolled in the Hispanic EPESE in 1993/94. Wave 4 (2000/01) through Wave 8 (2012/13) of the Hispanic EPESE data were linked with the Medicare Master Beneficiary Summary Files, Medicare Provider Analysis and Review (MedPAR) files, Outpatient Standard Analytic Files (OUTSAF) and Carrier files (1999 to 2012). The CMS matched our survey participants’ Social Security numbers with their Medicare beneficiary numbers. To ensure accuracy, the matched sample was further validated using additional variables: sex, date of birth, date of death and county of residence. This study only used part of the linked Hispanic EPESE-Medicare data. Healthcare use and Medicare payment information were retrieved from the Medicare claims files for a 12-month period after the Wave 4 assessment date (2000/01). We selected Wave 4 instead of earlier waves because of the CMS data availability at the time of conducting this study. As the sample size decreased over time, we used Wave 4 to maximize the total sample. This is the earliest wave with linked Medicare claims and frailty transition data we could include. The data use agreement procedures were approved by the CMS and the study was examined by the Institutional Review Board (IRB#: 16–0014) at our institution.

### Cohort selection criteria

A total of 2580 participants with successful linkage of Hispanic EPESE survey and Medicare claims. We excluded participants who were newly recruited in the survey (*n* = 464), who died, lost to follow-up or refused to be interviewed (*n* = 349), or had incomplete frailty assessment data at Wave 3 (*n* = 342). From Wave 3 (1988/89) to Wave 4 (2000/01), we excluded 318 participants due to death (*n* = 121), were lost to follow-up or refused to be interviewed (*n* = 91), or had incomplete frailty assessment data at Wave 4 (*n* = 106). After Wave 4 interviews, 319 individuals were excluded due to death during a year follow-up period (*n* = 42), lack of 12-moth continuous enrollment in Medicare fee-for-service plans (Parts A and B) or enrolled in a Health Maintenance Organization (*n* = 277) (Appendix Fig. 1). Each included participant had CMS healthcare use and Medicare payment data for a 12-month follow-up period after Wave 4.

### Modified frailty phenotype

We used the modified Fried et al. Frailty Phenotype [37] (four components) to identify frailty status for each individual: weight loss, weakness, exhaustion and slow walking. We did not use the physical activity component because this information was not collected across all waves of the Hispanic EPESE survey. Each participant was categorized as non-frail (0 criterion), pre-frail (1 criterion) or frail (2+ criteria). Participants with measured weight loss of > 4.5 kg were identified as having unintentional weight loss (score = 1). Grip strength (weakness) was assessed with a handheld dynamometer.^1^ Participants in the lower 20th percentile or those unable to perform the handgrip test were identified as positive for weakness (score = 1), after adjustment for sex and Body Mass Index (BMI) [calculated by weight (kg)/height (meters)]. Two items from the Center for Epidemiologic Studies Depression (CES-D) Scale [38] were used to assess exhaustion: “I felt everything I did was an effort” and “I could not get going”. The participant responded to “how often in the last week did you feel this way?” with a rating scale from 0 to 3 [0 = rarely or none (< 1 day), 1 = some or a little (1–2 days), 2 = a moderate amount of the time (3–4 days) or 3 = most of the time (5–7 days)]. Participants were identified as positive for exhaustion (score = 1) if they answered “2” or “3” to either of the two items. Walking speed was assessed using a 2.4-m timed walking test, adjusted for height and sex. Participants who scored in the slowest 20th percentile or who were unable to do the test were identified as positive for slow walking (score = 1). This protocol for assessing physical frailty (Frailty Phenotype) has been used in numerous studies [9, 18, 39–41].

### Frailty transitions

Frailty transition was defined as a change in frailty status between Wave 3 (1998/99) and Wave 4 (2000/01). Participants were stratified into five groups: 1) remain non-frail, 2) remain pre-frail, 3) remain frail, 4) improve (pre-frail to non-frail, frail to non-frail, frail to pre-frail) and 5) worse (non-frail to pre-frail, non-frail to frail, pre-frail to frail).

### Outcome measures

The main outcomes were: a) healthcare utilization (hospitalization, emergency room [ER] admission, physician visit) and b) Medicare payment (total payment and outpatient payment) during the observation period (from the Wave 4 assessment date to 12 months after). There were 20 and 32.6% of participants with at least one hospitalization and at least one ER admission, respectively. The mean of the number of hospitalization and ER admission was zero, so we defined them as dichotomous variables to show the percent of subjects experiencing any hospitalization and ER admission (yes/no). Data on hospitalization were derived from the MedPAR files using an admission date within the observation period. ER admission was derived from the MedPAR and OUTSAF files with an ER charge greater than zero on revenue center codes 0450, 0451, 0452, 0456, 0459 (for emergency room use) or 0981 (professional fees-emergency room use). Physician visits were counted as a continuous variable and derived from the OUTSAF and Carrier files using Current Procedural Terminology codes 99,201–99,205 (office or other outpatient visit for the evaluation and management of a new patient, requiring three components with different levels of complexity: patient history, patient examination and medical decision making) or 99,211–99,215 (under established patient office or other outpatient services; these codes were used to bill for patients who were treated in an office environment for either medical and/or mental health conditions). For Medicare payment, total payment included payment for acute short stays, inpatient rehabilitation facility stays, outpatient and skilled nursing facility stays. Data were retrieved from the MedPAR file and outpatient payment file. Outpatient payment included payment for any outpatient service from the OUTSAF and Carrier files, including evaluation and management (99201–99,499), pathology and laboratory (80047–89,398), medicine (90281–99,607), radiology (70010–79,999) and surgery (10021–69,990). Payments were adjusted to 2017 dollars using the medical care component of the Consumer Price Index [42].

### Covariates

Covariates included Wave 4 sociodemographic variables (age, sex, marital status and years of education), BMI, limitation in one or more activities of daily living (ADL); including walking, bathing, grooming, dressing, eating, transferring-bed to chair and toileting (yes/no), cognitive function assessed with the Mini-Mental State Examination (MMSE) [43] and comorbidity (self-reported presence of any of the following medical conditions: arthritis, diabetes, hypertension, heart attack, stroke, cancer or hip fracture) (yes/no).

### Statistical analyses

We used Chi-square, t-test and analysis of variance to examine the distributions of demographics, healthcare use and Medicare payment by included and excluded participants and by the five frailty transition groups. Logistic regression models were used to estimate the odds ratio (OR) of hospitalization and ER admission and zero-inflated negative binomial regression models were applied to estimate the rate ratio (RRs) of physician visit as a function of frailty transition status, controlling for all covariates. For Medicare payment, cost ratios (CRs) were estimated using generalized linear models with log-link gamma distribution. We extended the findings of previous studies [44, 45] by examining the interaction terms between five frailty transition groups and sex on the outcomes. The interaction terms were statistically significant between sex and hospitalization and outpatient payment (both *p* < 0.01). We thus further stratified our analysis by sex for hospitalization and outpatient payment. All analyses were conducted using SAS statistical software (version 9.4; SAS Institute Inc., Cary, NC) with a significance level of *p* < 0.05.

## Results

### Demographics

At Wave 4 (*N* = 788), participant mean age was 78.8 years (SD = 5.1), 60.2% were female and 47.2% were married. Mean years of education was 4.6 years (SD = 3.8), mean BMI was 28.1 kg/m^2^ (SD = 5.4) and mean MMSE was 22.1 (SD = 5.5). Thirty-seven percent of the participants (*n* = 288) had cognitive impairment (MMSE < 21) and 85.0% had at least one comorbid condition (Table 1). Compared to the study sample, the excluded sample (*n* = 319) was more male, more married, with higher education, higher MMSE, less cognitive impairment and higher CES-D (all *p* < 0.05) (Appendix Table 1). There were 52 (6.6%) participants with $0 total Medicare payment. Among 736 participants with payments greater than zero, only 14 participants (1.8%) did not have any hospitalization, ER admission or physician visit.

**Table 1:** Participant characteristics for the five frailty transition groups (*N* = 788)

Characteristics	Study Population		Frailty Transition Groups				
	Wave 3	Wave 4	Remain Non-frail	Improve^**#**^	Remain Pre-frail	Remain Frail	Worse^**^**^
No. of Subject	*N* = 1425	788	184	191	103	41	269
Age, years ^+^*	77.2 (5.5)	78.8 (5.1)	77.6 (4.4)	79.0 (5.2)	79.5 (5.4)	81.3 (6.0)	78.8 (5.0)
Female ^‡^	827 (58.1%)	474 (60.2%)	108 (58.7%)	117 (61.3%)	63 (61.2%)	26 (63.4%)	160 (59.5%)
US Born ^‡^	829 (58.2%)	459 (58.2%)	116 (63.0%)	106 (55.5%)	54 (52.4%)	27 (65.9%)	156 (58.0%)
Married ^‡^	736 (51.6%)	372 (47.2%)	96 (52.2%)	86 (45.0%)	44 (42.7%)	12 (29.3%)	134 (49.8%)
Education, years ^+^*	5.0 (3.9)	4.6 (3.8)	5.3 (4.1)	4.4 (3.7)	4.8 (3.4)	3.1 (2.7)	4.4 (3.7)
BMI, kg/m2 ^+^	28.3 (5.6)	28.1 (5.4)	28.0 (4.8)	28.7 (5.1)	28.3 (5.8)	28.9 (7.3)	27.5 (5.6)
ADL Total Score ^+*^	0.3 (1.1)	0.5 (1.4)	0.2 (1.0)	0.6 (1.5)	0.5 (1.5)	1.5 (2.1)	0.4 (1.2)
MMSE Total Score ^+^*	22.6 (5.5)	22.1 (5.5)	23.2 (4.7)	22.2 (5.5)	21.8 (5.5)	19.2 (5.8)	21.8 (5.7)
MMSE < 21 ^‡^*	473 (33.2%)	288 (36.7%)	52 (28.3%)	65 (34.0%)	39 (38.2%)	24 (58.5%)	108 (40.4%)
CES-D Total Score ^+^*	7.7 (8.6)	6.1 (6.9)	3.4 (4.2)	6.2 (5.9)	6.2 (6.3)	11.2 (9.6)	7.0 (8.0)
> = 1 Comorbidity ^‡^*	1033 (72.5%)	670 (85.0%)	145 (78.8%)	169 (88.5%)	89 (86.4%)	39 (95.1%)	228 (84.8%)
Hospitalization ^‡^*		161 (20.4%)	22 (12.0%)	40 (20.9%)	24 (23.3%)	14 (34.1%)	61 (22.7%)
ER admission ^‡^		257 (32.6%)	60 (32.6%)	55 (28.8%)	37 (35.9%)	17 (41.5%)	88 (32.7%)
Physician visit ^+^		9.0 (7.6)	8.1 (6.9)	9.0 (7.4)	8.5 (6.5)	9.5 (8.1)	9.9 (8.3)
Total payment ^&+^*		8609(19393)	5260(11970)	8794 (19114)	7674(16287)	10,204(14401)	10,883(24534)
Outpatient payment ^+^		3677(5332)	2774(3188)	4132 (7622)	3244(3286)	3676(3142)	4136(5366)
Outpatient service ^‡^							
Evaluation and Management *		725 (92.0%)	163 (88.6%)	171 (89.5%)	94 (91.3%)	39 (95.1%)	258 (95.9%)
Pathology and Laboratory		669 (84.9%)	155 (84.2%)	157 (82.2%)	81 (78.6%)	37 (90.2%)	239 (88.8%)
Medicine		641 (81.3%)	140 (76.1%)	155 (81.2%)	84 (81.6%)	36 (87.8%)	226 (84.0%)
Radiology		568 (72.1%)	128 (69.6%)	137 (71.7%)	73 (70.9%)	32 (78.0%)	198 (73.6%)
Surgery*		405 (51.4%)	84 (45.7%)	95 (49.7%)	55 (53.4%)	30 (73.2%)	141 (52.4%)

*Significant difference at *p*-value of < 0.05 among five transition groups. ^#^ Improved: pre-frail to non-frail; frail to non-frail; and frail to pre-frail. ^^^ Worse; non-frail to pre-frail; non-frail to frail; and pre-frail to frail. ^+^ Data was presented as mean (standard deviation); ^‡^ Data was presented as n(%). ^&^ A total of 52 (6.60%) participants with $0 total Medicare payment. Total payment includes acute short stays, inpatient rehabilitation facility stats, skilled nursing facility stays and outpatient services

***Abbreviations***: *BMI* body mass index, *ADL* activity of daily living, *IADL* instrumental activity of daily living, *MMSE* Mini-Mental State Examination, *CES-D* Center for Epidemiologic Studies Depression Scale

### Frailty transitions between wave 3 (1998/99) and wave 4 (2000/01)

Between Waves 3 and 4, 184 participants (23.4%) remained non-frail, 103 participants (13.1%) remained pre-frail, 41 participants (5.2%) remained frail, 191 (24.2%) transitioned to a better state (pre-frail to non-frail; frail to non-frail; and frail to pre-frail) and 269 participants (34.1%) transitioned to a worse state (non-frail to pre-frail; non-frail to frail; and pre-frail to frail) (Table 1). Overall, the majority of participants retained their frail status (41.6%). Appendix Table 2 provided more information on the specific transitions within the groups (e.g. numbers for each specific transition).

### Healthcare use and Medicare payment by transition groups

Participants in the five frailty transition groups differ significantly in their rates of hospitalization and Medicare total payment (both *p* < 0.05). Participants who remained frail had the highest rate of hospitalization (34.1%). Participants who transitioned to a worse status had the highest Medicare total payment ($10,883), similar to that of those who remained frail ($10,204). Participants who transitioned to a better status had a similar outpatient payment ($4132) to those who transitioned to a worse status ($4136). Participants who remained frail were significantly more likely to be older, have fewer years of education, more ADL disability, more cognitive impairment or depressive symptoms, and more comorbidities compared to those who remained non-frail (Table 1).

Table 2 reports the observed and adjusted ORs, RRs and CRs with 95% CI for healthcare use and Medicare payment as a function of being in one of the five frailty transition groups, after controlling for socio-demographics, BMI, ADL disability, cognitive impairment, depression and comorbidities. Due to missing data for education, ADL and the MMSE, the final sample included in the multivariate outcome model was 773. In the adjusted model, compared to the ‘remained non-frail’ group, the OR for hospitalization was 2.65 (95% CI = 1.13–6.24) for participants who remained frail, 1.97 (95% CI = 1.03–3.77) for those who remained pre-frail and 2.00 (95% CI = 1.17–3.43) for those who transitioned to a worse state of frailty. In participants who transitioned to a better frailty status, the OR for hospitalization (1.60, 95% CI = 0.89–2.87) was also higher than that for the non-frail group, but the difference was not statistically significant (Table 2).

**Table 2:** Effect of early frailty transition on healthcare utilization and Medicare payment (*N* = 773)

Transition Group	Hospitalization	ER Admission	Physician Visit	Total payment	Outpatient payment
	OR (95%CI)	OR (95%CI)	RR (95%CI)	CR (95%CI)	CR (95%CI)
Base Model (N = 788)					
Remained Non-frail	Reference	Reference	Reference	Reference	Reference
Improve	1.95 (1.11–3.43) *	0.84 (0.54–1.30)	1.12 (0.95–1.31)	1.67 (1.21–2.30) *	1.49 (1.14–1.95) *
Remained Pre-frail	2.24 (1.18–4.23) *	1.16 (0.70–1.92)	1.05 (0.87–1.28)	1.46 (1.00–2.14)	1.17 (0.85–1.61)
Remained Frail	3.82 (1.74–8.36) *	1.46 (0.73–2.93)	1.14 (0.88–1.49)	1.94 (1.14–3.31) *	1.33 (0.85–2.07)
Worse	2.16 (1.27–3.66) *	1.00 (0.67–1.50)	1.18 (1.02–1.37)	2.07 (1.54–2.78) *	1.49 (1.16–1.91) *
Multivariate (N = 773)^&^					
Remained Non-frail	Reference	Reference	Reference	Reference	Reference
Improve	1.60 (0.89–2.87)	0.72 (0.45–1.13)	1.07 (0.91–1.27)	1.51 (1.09–2.10) *	1.42 (1.08–1.87) *
Remained Pre-frail	1.97 (1.03–3.77) *	0.93 (0.55–1.58)	1.03 (0.84–1.25)	1.29 (0.88–1.89)	1.13 (0.82–1.56)
Remained Frail	2.65 (1.13–6.24) *	0.90 (0.41–1.95)	1.10 (0.83–1.46)	1.40 (0.79–2.48)	1.13 (0.70–1.81)
Worse	2.00 (1.17–3.43) *	0.89 (0.59–1.35)	1.17 (1.01–1.36)	2.09 (1.55–2.83) *	1.47 (1.15–1.89) *

*Significant difference at *p*-value of < 0.05 ^&^ Multivariate model: Adjusted for age, sex, marital status, education, body mass index, comorbidity, activities of daily living (ADL), Mini-Mental State Examination (MMSE). Due to missing data on education, ADL, and MMSE, the final sample included in the multivariate model is 773

***Abbreviations***: *OR* odds ratio, *ER* emergency department, *CI* confidence interval, *RR* rate ratio, *CR* cost ratio

Compared to participants who remained non-frail, those with improved frailty status had a CR 1.51 (95% CI = 1.09–2.10) times higher; participants who transitioned to a worse state had a CR 2.09 (95% CI = 1.55–2.83) times higher. A similar trend was found for Medicare outpatient payment CRs (1.42 [95% CI = 1.08–1.87] for the improved group and 1.47 [95% CI = 1.15–1.89] for the worse group) (Table 2).

Appendix Table 3 compares healthcare use and Medicare payment among specific frailty transition groups (worse vs. improved). Among the worsening groups, the transition from pre-frail to frail had a significant higher hospitalization than the non-frail to frail or the non-frail to pre-frail groups (*p* = 0.005). Among the improving groups, the transition from frail to pre-frail had a significantly higher outpatient payment than the pre-frail to non-frail or frail to non-frail groups (*p* = 0.030).

### Sex difference in outcomes by frailty transition group

Appendix Table 4 presents sample distributions of five transition groups by sex for five outcomes (hospitalization, ER admission, physician visit, total Medicare payment and outpatient Medicare payment). Among individuals who improved their frailty status, females were significantly more likely to be U.S. born and unmarried; and they had less ADL impairment, more physician visits and higher outpatient payments. Females also used more outpatient, pathology/laboratory, medicine, radiology and surgery services than males (all *p* < 0.05). Similar results by sex were found in the remaining non-frail and worsening frail groups.

Table 3 reports the adjusted ORs and CRs with 95% CI for hospitalization and Medicare outpatient payment as a function of frailty transition status stratified by sex and controlling for all covariates. Hospitalization and outpatient payment are the only two outcomes with significant interaction effect on sex. Compared to participants who remained non-frail, the OR for hospitalization was 3.49 (95% CI = 1.13–10.8) for those who remained pre-frail, 6.92 (95% CI = 1.61–29.7) for those who remained frail and 4.49 (95% CI = 1.74–11.6) for those who transitioned to a worse frailty status among males. However, similar trends were not found in females (Table 3).

**Table 3:** Outcomes with significant interaction effect on sex: Hospitalization and Medicare outpatient payment (*N* = 773)

Transition Group	Hospitalization		Outpatient payment	
	Male	Female	Male	Female
Base Model	OR	OR	CR	CR
Remained Non-frail	Reference	Reference	Reference	Reference
Improve	2.04 (0.71–5.83)	1.89 (0.96–3.73)	1.13 (0.71–1.80)	1.63 (1.20–2.23) *
Remained Pre-frail	3.89 (1.30–11.7) *	1.64 (0.74–3.64)	1.49 (0.85–2.61)	1.01 (0.70–1.46)
Remained Frail	7.78 (2.06–29.3) *	2.56 (0.95–6.86)	1.65 (0.73–3.70)	1.16 (0.70–1.94)
Worse	4.85 (1.91–12.3) *	1.27 (0.65–2.48)	2.08 (1.36–3.19) *	1.20 (0.90–1.61)
Multivariate				
Remained Non-frail	Reference	Reference	Reference	Reference
Improve	1.93 (0.65–5.75)	1.54 (0.76–3.12)	1.14 (0.70–1.85)	1.53 (1.12–2.09) *
Remained Pre-frail	3.49 (1.13–10.8) *	1.44 (0.63–3.26)	1.40 (0.79–2.48)	0.96 (0.66–1.39)
Remained Frail	6.92 (1.61–29.7) *	1.45 (0.48–4.34)	1.49 (0.61–3.63)	0.92 (0.54–1.57)
Worse	4.49 (1.74–11.6) *	1.17 (0.59–2.33)	2.00 (1.30–3.09) *	1.16 (0.87–1.56)

*Significant difference at *p*-value of < 0.05

OR: Odds Ratio. CR: Cost Ratio; Multivariate model: adjusted for age, marital status, education, BMI, comorbidity, ADL, MMSE. ***Abbreviations***: *BMI* body mass index, *ADL* activities of daily living, *MMSE* Mini-Mental State Examination

Compared to males who remained non-frail, males who transitioned to a worse status had 2.00 (95% CI = 1.30–3.09) greater odds of higher Medicare outpatient payments. Compared to females who remained non-frail, females with frailty improvement had 1.53 (95% CI = 1.12–2.09) greater odds of higher Medicare outpatient payment (Table 3).

## Discussion

Using linked Medicare claims and Hispanic EPESE survey data, we examined change and/or stability in frailty status related to the use of healthcare services and Medicare payment in older Mexican Americans. We also examined these associations for males versus females. Our results indicate that participants who remained pre-frail and those who either remained frail or become more frail during the one-year study period experienced a higher risk of hospitalization. However, this association was statistically significant only in males.

Females whose frailty status improved, or who remained non-frail, had more physician and outpatient visits, and used more radiology, surgery and laboratory services compared to males. These findings may explain the higher Medicare outpatient payments associated with frailty deterioration in males, but associated with frailty improvement in females. Our findings suggest that care-seeking behaviors may be influenced by sex and lead to different health risks and levels of payment for older Mexican American who are living in the community. These findings have implications for biological and psycho-social factors associated with changes in frailty status and the use of healthcare services in Mexican American males versus females older adults.

Transitioning to a less frail status would logically improve function and quality of life for patients, but doing so may require additional attention and care, and therefore more healthcare services, resulting in higher Medicare payments. Our findings suggest that improvement to a less frail status (e.g., pre-frail or non-frail) is possible, but how to put the required increasing medical and care resources in place is a question. This is an important area for future research, particularly in the current climate of value-based purchasing with more emphasis on patient centered quality metrics [46, 47]. Particularly for older Mexican Americans, additional efforts and design may be needed to ensure this population can receive care resources they need. We also found the transitions from pre-frail to frail (and vice versa) played a superior role in follow-up hospitalization and outpatient payment. The study findings partially support our hypothesis as we found being more frail associated with more healthcare use and Medicare payment. However, even if the transitions occurred in the same worsening or improving directions, the frailty transitions required different levels of follow-up healthcare resources in older Mexican Americans.

The higher frailty prevalence among minority populations is an important reason to perform this study. Identifying factors associated with changes in frailty status is important in developing models of value-based care and preventive services for older Mexican Americans. Pre-frail is a middle phase that can move into a worsening or improving frailty status. We suggest primary care providers conducting frailty screening to identify older Mexican Americans as pre-frail early [48, 49]. Mexican-origin Latinos in California were found having lower rates of primary care use than non-Latino White or the second generations [50]. These individuals are potential candidates who may benefit the most from the early pre-frail identification and receive low-cost frailty preventive programs (e.g., nutritional counselling and group exercise programs such as Tai Chi) [51]. Our findings also highlight the need to consider sex-specific frailty programs and activities for the older Mexican Americans living in the community. Future research is necessary to determine if frailty prevention and intervention programs should be targeted by sex for persons identified as pre-frail in other racial and ethnic groups.

### Study limitations

The generalizability of our finding is limited due to the exclusion criteria and the fact that only claims data from Medicare beneficiaries with fee-for-service coverage were available from CMS. We did not include control group (other race/ethnicity) in this study. As a result, it is unclear whether our findings are relevant to the development of treatment for frailty interventions, or whether our findings can apply to all older adults. Using the modified Frailty Phenotype (four instead of the five components in the original measure [26]) may potentially decrease the sensitivity of the frailty assessment. The Frailty Phenotype used in our study is a measure of physical frailty [37] and does not include information related to cognitive function.

An alternate measure of frailty, the Frailty Index, uses cumulative deficits such as disabilities, diseases and other signs and symptoms that may provide a greater degree of sensitivity in identifying frailty transitions [52]. However, the Hispanic EPESE does not have the required clinical and medical data to construct the Frailty Index. The impact of frailty transitions beyond the observed period is also unknown as our outcome was limited to 1 year after the frailty transition classifications were identified. The sample size in the group that remained frail (*n* = 41) was small. We considered combining this smaller group with other groups. However, this action would have reduced our ability to maintain the focus on persons who experienced consistently frail status during the period studied. Lastly, we studied payments instead of cost in this study. We acknowledged that Medical payments are not equivalent to the healthcare use and covers broader areas than healthcare use as defined in this study.

## Conclusion

We found that changes in frailty status in older Mexican Americans are associated differently with subsequent hospitalizations and Medicare payments for outpatient health care services. Compared to persons who remained non-frail, males in all transition groups except one had higher risks of hospitalization. Females transitioning to improved frailty status had higher Medicare outpatient payment for outpatient services; this pattern did not occur in males. We found consistent differences between males and females in the patterns of frailty transitions associated with the use of healthcare resources and Medicare payment for outpatient services. Differences and disparities between Mexican Americans and other racial and ethnic groups have been widely reported [2, 4]. Our findings expand our understanding that there may be differences based on sex (male/female) related to the development, treatment and prevention of frailty in older Mexican Americans and the importance of pre-frail state for frailty recover/advancement. Whether these differences are disparities is a question that deserves additional scientific attention.

## Figures and Tables

**Fig. 1:**
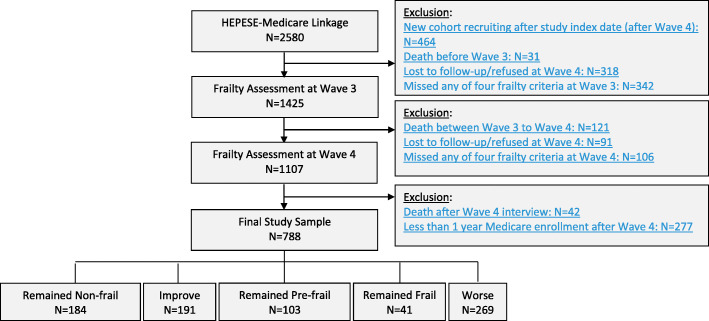
Cohort selection diagram

## Data Availability

Hispanic-EPESE data are publicly available online through the National Archive of Computerized Data on Aging, located at the Inter-university Consortium for Political and Social Research managed by the University of Michigan at Ann Arbor (https://www.icpsr.umich.edu/icpsrweb/NACDA/series/00546). Medicare claims are from the Centers for Medicare and Medicaid Services (CMS) and are not publicly available based on the Data Use Agreement (DUA) with CMS. The CMS data used in the study are available from CMS Research Data Assistance Center (ResDAC) (https://www.resdac.org/).

## Appendix A

**Table 4:** Comparisons of characteristics between the study sample (*n* = 788) and the excluded sample (*n* = 319)

Characteristics at Wave 4	Study Sample (n = 788)	Excluded Sample (n = 319)	*P*-value
Age, mean (SD), years	78.8 (5.1)	78.7 (4.9)	0.767
Female, n(%)	474 (60.2%)	167 (52.4%)	0.017*
US Born, n(%)	459 (58.2%)	181 (56.7%)	0.645
Married, n(%)	372 (47.2%)	184 (57.7%)	0.002*
Education, years, mean (SD)	4.6 (3.8)	5.9 (4.0)	< 0.001*
BMI, kg/m2, mean (SD)	28.1 (5.4)	27.7 (5.4)	0.271
ADL, mean (SD)	0.5 (1.4)	0.4 (1.1)	0.109
MMSE, mean (SD)	22.1 (5.5)	23.4 (5.0)	< 0.001*
MMSE < 21, n(%)	288 (36.7%)	87 (27.3%)	0.003*
CES-D, mean (SD)	6.1 (6.9)	7.1 (7.8)	0.036*
> = 1 Comorbidity	670 (85.0%)	272 (85.3%)	0.919

***Abbreviations***: *SD* standard deviation, *BMI* body mass index, *ADL* activities of daily living, *IADL* instrumental activities of daily living, *MMSE* Mini-Mental State Examination, *CES-D* Center for Epidemiologic Studies Depression Scale

## Appendix B

**Table 5:** Distribution of nine frailty transitions from Wave 3 to Wave 4

Transition Groups	N	Frailty Status		Male	Female
		Wave 3	Wave 4	n (%)	n (%)
Remained Non-frail	184	Non-frail	Non-frail	76 (41.3%)	108 (58.7%)
Remained Pre-frail	103	Pre-frail	Pre-frail	40 (38.8%)	63 (61.2%)
Remained frail	41	Frail	Frail	15 (36.6%)	26 (63.4%)
Improve	102	Pre-frail	Non-frail	41 (40.2%)	61 (59.8%)
	33	Frail	Non-frail	12 (36.4%)	21 (63.6%)
	56	Frail	Pre-frail	21 (37.5%)	35 (62.5%)
Worse	155	Non-frail	Pre-frail	68 (43.9%)	87 (56.1%)
	55	Non-frail	Frail	15 (27.3%)	40 (72.7%)
	59	Pre-frail	Frail	26 (44.1%)	33 (55.9%)

## Appendix C

**Table 6:** Comparisons of healthcare utilization and Medicare payment among frailty transition (worse vs. improved)

Worse frailty transitions				
	Non-frail to Pre-frail	Non-frail to frail	Pre-frail to frail	p-value
	*N* = 155	*N* = 55	*N* = 59	
Hospitalization	32 (20.7%)	*N* < 11	22 (37.3%)	0.005*
ER admission	48 (31.0%)	18 (32.7%)	22 (37.3%)	0.679
Physician visit	9.4 (7.9)	9.2 (6.6)	11.7 (10.3)	0.145
Total payment	8688 (18737)	10,456 (33388)	17,044 (27563)	0.083
Outpatient payment	3748 (5616)	3901 (4722)	5375 (5159)	0.131
**Improved frailty transitions**				
	Pre-frail to Non-frail	Frail to Non-frail	Frail to Pre-frail	p-value
	*N* = 102	*N* = 33	*N* = 56	
Hospitalization	17 (16.7%)	N < 11*	15 (26.8%)	0.287
ER admission	24 (23.5%)	N < 11	22 (39.3%)	0.110
Physician visit	8.4 (7.7)	10.3 (7.2)	9.4 (6.8)	0.360
Total payment	6322 (15584)	9665 (18571)	12,784 (24238)	0.121
Outpatient payment	2986 (4942)	3954 (4160)	6327 (11766)	0.030*

* We presented the sample size less than 11 participants as *N* < 11 due to CMS cell size suppression policy (see: https://www.resdac.org/articles/cms-cell-size-suppression-policy)

## Appendix D

**Table 7:** Participant characteristics by frailty transition group and sex (*N =* 788)

Variable	Frailty Transition Groups									
	Remained Non-frail		Improve^**#**^		Remained Pre-frail		Remained Frail		Worse^**^**^	
	Male	Female	Male	Female	Male	Female	Male	Female	Male	Female
No. of Subject	76	108	74	117	40	63	15	26	109	160
Age, years ^+^	78.1 (4.3)	77.2 (4.4)	79.2 (5.1)	79.0 (5.2)	79.3 (5.0)	79.7 (5.6)	80.8 (5.8)	81.6 (6.3)	78.5 (4.9)	79.0 (5.1)
US Born ^‡^*	44 (57.9%)	72 (66.7%)	32 (43.2%)	**74 (63.2%)**	22 (55.0%)	32 (50.8%)	8 (53.3%)	19 (73.1%)	55 (50.5%)	**101 (63.1%)**
Married ^‡^*	60 (78.9%)	**36 (33.3%)**	44 (59.5%)	**42 (35.9%)**	27 (67.5%)	**17 (27.0%)**	7 (46.7%)	5 (19.2%)	84 (77.1%)	**50 (31.3%)**
Education, years ^+^	5.2 (4.0)	5.4 (4.2)	3.8 (3.2)	4.8 (4.0)	4.6 (3.5)	4.9 (3.3)	2.6 (2.9)	3.3 (2.6)	4.5 (4.0)	4.4 (3.5)
BMI, kg/m2 ^+^*	26.9 (4.1)	**28.8 (5.1)**	28.1 (4.4)	29.0 (5.5)	27.6 (5.2)	28.7 (6.1)	26.2 (5.3)	30.4 (7.9)	26.8 (4.0)	28.0 (6.5)
ADL Total Score ^+^*	0.1 (0.4)	0.3 (1.3)	0.3 (1.1)	**0.7 (1.7)**	0.4 (1.3)	0.6 (1.6)	1.1 (1.9)	1.8 (2.2)	0.2 (1.0)	**0.6 (1.4)**
MMSE Total Score ^+^	23.2 (4.7)	23.2 (4.8)	22.5 (5.0)	22.1 (5.8)	21.3 (5.4)	22.1 (5.7)	19.3 (6.2)	19.2 (5.6)	21.2 (5.8)	22.2 (5.5)
MMSE < 21 ^‡^	23 (30.3%)	29 (26.9%)	24 (32.4%)	41 (35.0%)	15 (37.5%)	24 (38.7%)	N/A	15 (57.7%)	52 (48.1%)	**56 (35.2%)**
CES-D (Total) ^+^*	2.7 (3.7)	3.9 (4.4)	5.2 (5.2)	6.8 (6.3)	5.3 (5.4)	6.7 (6.8)	8.7 (7.4)	12.7 (10.5)	5.8 (7.3)	**7.8 (8.4)**
> = 1 Comorbidity ^‡^*	54 (71.1%)	**91 (84.3%)**	62 (83.8%)	107 (91.5%)	34 (85.0%)	55 (87.3%)	13 (86.7%)	26 (100.0%)	91 (83.5%)	137 (85.6%)
Hospitalization ^‡^	N/A	16 (14.8%)	11 (14.9%)	29 (24.8%)	N/A	14 (22.2%)	N/A	N/A	32 (29.4%)	**29 (18.1%)**
ER admission ^‡^*	14 (18.4%)	**46 (42.6%)**	17 (23.0%)	38 (32.5%)	13 (32.5%)	24 (38.1%)	N/A	N/A	32 (29.4%)	56 (35.0%)
Physician visit ^+^*	6.7 (7.7)	**9.0 (6.2)**	6.9 (6.8)	**10.3 (7.5)**	7.9 (5.7)	9.0 (7.0)	7.8 (6.3)	10.5 (9.0)	9.7 (7.8)	10.0 (8.7)
Total payment^&+^	4007.9 (9567.3)	6141.3 (13,379.4)	8362.1 (19,657.1)	9067.3 (18,842.9)	6239.0 (9853.9)	8584.2 (19,317.4)	11,409.2 (14,248.9)	9508.8 (14,723.1)	13,263.5 (28,690.8)	9260.0 (21,187.9)
Outpatient payment^+^	2240.4 (2726.3)	3149.4 (3438.9)	2525.9 (3751.9)	**5148.5 (9144.2)**	3344.8 (2892.1)	3179.3 (3533.6)	3692.3 (2764.2)	3666.6 (3393.9)	4661.3 (4791.7)	3778.8 (5711.1)
Outpatient service ^‡^										
Evaluation and Management *	58 (76.3%)	**105 (97.2%)**	59 (79.7%)	**112 (95.7%)**	37 (92.5%)	57 (90.5%)	14 (93.3%)	25 (96.2%)	101 (92.7%)	157 (98.1%)
Pathology and Laboratory *	55 (72.4%)	**100 (92.6%)**	49 (66.2%)	**108 (92.3%)**	32 (80.0%)	49 (77.8%)	14 (93.3%)	23 (88.5%)	93 (85.3%)	146 (91.3%)
Medicine *	48 (63.2%)	**92 (85.2%)**	48 (64.9%)	**107 (91.5%)**	30 (75.0%)	54 (85.7%)	15 (100.0%)	21 (80.8%)	89 (81.7%)	137 (85.6%)
Radiology *	42 (55.3%)	**86 (79.6%)**	42 (56.8%)	**95 (81.2%)**	27 (67.5%)	46 (73.0%)	13 (86.7%)	19 (73.1%)	75 (68.8%)	123 (76.9%)
Surgery	30 (39.5%)	54 (50.0%)	29 (39.2%)	**66 (56.4%)**	26 (65.0%)	29 (46.0%)	12 (80.0%)	18 (69.2%)	63 (57.8%)	78 (48.8%)

* Significant difference at p-value of < 0.05 across five transition groups. Bold for significant difference at p-vale of < 0.05 within the same transition group. ^+^ Data was presented as mean (standard deviation); ^‡^ Data was presented as N(%) ^&^ There are 52 (6.59%) participants with $0 total Medicare payment. Total payment includes acute short stays, inpatient rehabilitation facility stats, skilled nursing facility stays and outpatient services

***Abbreviations***: *BMI* body mass index, *ADL* activity of daily living, *MMSE* Mini-Mental State Examination, *CES-D* Center for Epidemiologic Studies Depression Scale. ^#^ Improved: pre-frail to non-frail; frail to non-frail; and frail to pre-frail. ^^^ Worse; non-frail to pre-frail; non-frail to frail; and pre-frail to frail

## Abbreviations

ADL: Activities of Daily Living
BMI: Body Mass Index
CES-D: Center for Epidemiologic Studies Depression Scale
ER: Emergency room
HR: Hazards ratios
Hispanic-EPESE: Hispanic Established Populations for the Epidemiological Study of the Elderly
MedPAR: Medicare Provider Analysis and Review
MMSE: Mini-Mental State Examination
OUTSAF: Outpatient Standard Analytic Files
SD: Standard Deviation

## Footnotes

1 Jaymar Hydraulic Dynamo-meter model #5030 J1; J.A. Preston Corp., Jackson, MI
